# Supplementary material for: Two-Dimensional Transition Metal-Hexaaminobenzene Monolayer Single-Atom Catalyst for Electrocatalytic Carbon Dioxide Reduction
Source: Nanomaterials (Basel). 2022 Nov 14;12(22):4005. doi: 10.3390/nano12224005 (PMC9693506; doi:10.3390/nano12224005)
Supplement: Supplementary file 1 [file nanomaterials-12-04005-s001.zip › nanomaterials-2002097-supplementary.pdf]

Supplementary Materials

Two-Dimensional Transition Metal-Hexaaminobenzene Monolayer Single-Atom Catalyst for Electrocatalytic Carbon Dioxide Reduction

Table S1. Lattice constant a and b of TM−HAB in Å, where TM are the metal atoms of the first transition metal series.

TM−HAB	Lattice(a)	Lattice(b)
Sc-HAB	14.000	14.000
Ti-HAB	13.648	13.731
V-HAB	13.924	13.746
Cr-HAB	13.830	13.782
Mn-HAB	14.765	13.274
Fe-HAB	14.000	14.316
Co-HAB	14.550	13.279
Ni-HAB	13.419	13.417
Cu-HAB	13.695	13.761
Zn-HAB	14.014	13.997

Table S2. E_c is the cohesive energy of the bulk TM, E_b is the binding energy between the TM and the TM–HAB, and E_f is the formation energy of TM–HAB, where TM are the metal atoms of the first transition metal series.

TM-HAB	Eb(eV)	Ec(eV)	Ef(eV)
TM-Sc	-12.049	-4.423	-7.877
TM-Ti	-13.309	-5.956	-6.285
TM-V	-11.967	-6.578	-5.316
TM-Cr	-9.710	-4.011	-5.169
TM-Mn	-10.524	-3.761	-4.706
TM-Fe	-10.476	-5.264	-5.122
TM-Co	-11.181	-6.051	-3.831
TM-Ni	-10.928	-6.017	-4.442
TM-Cu	-6.991	-3.431	-2.587
Zn	-5.379	-1.054	-3.871

(1)E_b is binding energy of metal atoms on the HAB, which is calculated by:

$$E_b = E_{TM-HAB} - E_{TM} - E_{HAB},$$

where the E_{TM-HAB}, E_{HAB} and E_{TM} are the energies of TM-HAB, HAB and metal atoms.
(2)Ec is the cohesive energy of metals, which is calculated by:

$$E_c = (E_{M(bulk)} - nE_M)/n$$

where the $E_{M(bulk)}$ is the energy of metal crystal, E_M is the energy of single metal atoms and n is the number of metal atoms in the crystal.
(3)E_f is the formation energy of TM-HAB, which is calculated by

$$E_f = E_{TM-HAB} - n_{TM}\mu_{TM} - n_C\mu_C - n_N\mu_N - n_H\mu_H$$

where the E_{TM-HAB} are the energies of TM-HAB, n_{TM}, n_C, n_N and n_H are the number of TM, C, N and H, respectively. μ_{TM}, μ_C, μ_N and μ_H arethe chemical potential of TM, C, N and H, respectively.

Table S3. Gibbs free energy change (ΔG/eV) of the first protonation step in the CO_2 reduction reaction (CO_2RR) and H_2 evolution reaction (HER) on the TM–HAB.

TM-HAB	ΔG[*H]	ΔG[*COOH]	ΔG[*OCHO]
Sc-HAB	0.061	-0.526	-2.366
Ti-HAB	-0.496	-0.726	-2.126
V-HAB	-0.373	-0.803	-0.713
Cr-HAB	-0.247	-0.572	-1.344
Mn-HAB	0.118	-0.263	-0.826
Fe-HAB	0.011	-0.351	-0.652
Co-HAB	-0.410	-0.865	-0.995
Ni-HAB	-1.058	-1.277	-2.039
Cu-HAB	0.741	0.162	-0.742
Zn-HAB	0.996	-0.066	-1.398

Table S4. Gibbs free energy change for each protonation step of Zn-HAB electrocatalytic CO_2 reduction.

n(H^+ + e^-)transferred	Chemical reaction equation	ΔG
1	* + CO_2 + H^+ + e^- →*COOH	0.933
	* + CO_2 + H^+ + e^- →*OCHO	-0.398
2	*OCHO + H^+ + e^- →*OCHOH	-0.061
3	*OCHOH + H^+ + e^- → *CHO + H_2O	1.314
	*OCHOH + H^+ + e^- →*OCH + H2O	1.291
	OCHOH→+HCOOH	0.243

Table S5. Gibbs free energy change for each protonation step of Co-HAB electrocatalytic CO_2 reduction.

n(H^+ + e^-)transferred	Chemical reaction equation	ΔG
1	* + CO_2 + H^+ + e^- → *COOH	-0.865
	* + CO_2 + H^+ + e^- → *OCHO	-0.995
2	*COOH + H^+ + e^- → *CO + H_2O	-0.064
	*OCHO + H^+ + e^- → *OCHOH	2.630
3	*CO + H_2O + H^+ + e^- → *CHO + H_2O	-0.226
	*CO + H_2O + H^+ + e^- → *COH + H_2O	1.732
4	*CHO + H_2O + H^+ + e^- → *OCH_2 + H_2O	0.532
5	*OCH_2 + H_2O + H^+ + e^- → *OCH_3 + H_2O	-0.709
6	*OCH_3 + H_2O + H^+ + e^- → *CH_3OH + H_2O	-0.475
	*OCH_3 + H_2O + H^+ + e^- → *O + CH_4 +H_2O	-0.081
7	*O + CH_4 + H_2O + H^+ + e^- → *OH + CH_4 + H_2O	-1.148
	*CH_3OH + H_2O + H^+ + e^- → *OH + CH_4 + H_2O	-0.754
	*CH_3OH + H_2O → * + CH_3OH + H_2O	0.465
8	*OH + CH_4 + H_2O + H^+ + e^- → * + CH_4 + $2H_2O$	0.095

Table S6. Gibbs free energy change for each protonation step of Mn-HAB electrocatalytic CO_2 reduction.

$n(H^+ + e^-)$transferred	Chemical reaction equation	ΔG
1	$* + CO_2 + H^+ + e^- \rightarrow *COOH$	-0.263
	$* + CO_2 + H^+ + e^- \rightarrow *OCHO$	-0.826
2	$*COOH + H^+ + e^- \rightarrow *CO + H_2O$	-0.555
	$*OCHO + H^+ + e^- \rightarrow *OCHOH$	-0.307
3	$*CO + H_2O + H^+ + e^- \rightarrow *CHO + H_2O$	0.278
	$*CO + H_2O + H^+ + e^- \rightarrow *COH + H_2O$	1.601
	$*OCHOH + H^+ + e^- \rightarrow *CHO + H_2O$	0.593
	$*OCHOH + H^+ + e^- \rightarrow *OCH + H_2O$	2.190
4	$*CHO + H_2O + H^+ + e^- \rightarrow *OCH_2 + H_2O$	-0.078
5	$*OCH_2 + H_2O + H^+ + e^- \rightarrow *OCH_3 + H_2O$	-0.909
	$*OCH_2 + H_2O + H^+ + e^- \rightarrow * + HCHO + H_2O + H^+ + e^-$	0.231
6	$*OCH_3 + H_2O + H^+ + e^- \rightarrow *CH_3OH + H_2O$	-0.227
	$*OCH_3 + H_2O + H^+ + e^- \rightarrow *O + CH_4 + H_2O$	-0.572
7	$*O + CH_4 + H_2O + H^+ + e^- \rightarrow *OH + CH_4 + H_2O$	-0.795
	$*CH_3OH + H_2O + H^+ + e^- \rightarrow *OH + CH_4 + H_2O$	-1.141
	$*CH_3OH + H_2O \rightarrow * + CH_3OH + H_2O$	0.273
8	$*OH + CH_4 + H_2O + H^+ + e^- \rightarrow * + CH_4 + 2H_2O$	0.143

Table S7. Gibbs free energy change for each protonation step of Fe-HAB electrocatalytic CO_2 reduction.

$n(H^+ + e^-)$transferred	Chemical reaction equation	ΔG
1	$* + CO_2 + H^+ + e^- \rightarrow *COOH$	-0.351
	$* + CO_2 + H^+ + e^- \rightarrow *OCHO$	-0.653
2	$*COOH + H^+ + e^- \rightarrow *CO + H_2O$	-0.350
	$*OCHO + H^+ + e^- \rightarrow *OCHOH$	0.514
3	$*CO + H_2O + H^+ + e^- \rightarrow *CHO + H_2O$	0.101
	$*CO + H_2O + H^+ + e^- \rightarrow *COH + H_2O$	1.806
4	$*CHO + H_2O + H^+ + e^- \rightarrow *OCH_2 + H_2O$	0.274
5	$*OCH_2 + H_2O + H^+ + e^- \rightarrow *OCH_3 + H_2O$	-0.825
	$*OCH_2 + H_2O + H^+ + e^- \rightarrow * + HCHO + H_2O + H^+ + e^-$	0.102
6	$*OCH_3 + H_2O + H^+ + e^- \rightarrow *CH_3OH + H_2O$	-0.020
	$*OCH_3 + H_2O + H^+ + e^- \rightarrow *O + CH_4 + H_2O$	-0.329
7	$*O + CH_4 + H_2O + H^+ + e^- \rightarrow *OH + CH_4 + H_2O$	-0.778
	$*CH_3OH + H_2O + H^+ + e^- \rightarrow *OH + CH_4 + H_2O$	-1.088
	$*CH_3OH + H_2O \rightarrow * + CH_3OH + H_2O$	0.098
8	$*OH + CH_4 + H_2O + H^+ + e^- \rightarrow * + CH_4 + 2H_2O$	-0.016

Table S8. Gibbs free energy change for each protonation step of Ti-HAB electrocatalytic CO_2 reduction.

n(H^++ e^-)transferred	Chemical reaction equation	ΔG
1	* + CO_2 + H^+ + e^- → *COOH	-1.726
	* + CO_2 + H^+ + e^- → *OCHO	-2.126
2	*COOH + H^+ + e^- →*CO + H_2O	0.569
	*OCHO + H^+ + e^- → *OCHOH	-1.149
3	*CO + H_2O + H^+ + e^- → *CHO + H_2O	-0.363
	*CO + H_2O + H^+ + e^- → *COH + H_2O	1.127
	*OCHOH + H^+ + e^- →*CHO + H_2O	1.755
	*OCHOH + H^+ + e^- → *OCH + H_2O	2.523
4	*CHO + H_2O + H^+ + e^- → *OCH_2 + H_2O	-0.707
	*COH + H_2O + H^+ + e^- →*C + 2H_2O	2.125
5	*OCH_2 + H_2O + H^+ + e^- → *OCH_3 + H_2O	0.313
6	*OCH_3 + H_2O + H^+ + e^- → *CH_3OH + H_2O	0.193
	*OCH_3 + H_2O + H^+ + e^- →*O + CH_4 + H_2O	-1.780
7	*O + CH_4 + H_2O + H^+ + e^- → *OH + CH_4 + H_2O	0.084
	*CH_3OH + H_2O + H^+ + e^- → *OH + CH_4 + H_2O	-1.889
8	*OH + CH4 + H_2O + H^+ + e^-→ * + CH_4 + 2H_2O	1.140

Table S9. Gibbs free energy change for each protonation step of Sc-HAB electrocatalytic CO_2 reduction.

n(H^++e^-)transferred	Chemical reaction equation	ΔG
1	* + CO_2 + H^+ + e^- → *COOH	-0.445
	* + CO_2 + H^+ + e^- → *OCHO	-2.285
2	*COOH + H^+ + e^- → *CO + H_2O	0.071
	*OCHO + H^+ + e^-→ *OCHOH	-0.157
3	*CO + H_2O + H^+ + e^-→ *CHO + H_2O	0.223
	*CO + H_2O + H^+ + e^- → *COH + H_2O	1.489
	*OCHOH + H^+ + e^- → *CHO + H_2O	2.292
	*OCHOH + H^+ + e^- → *OCH + H_2O	2.710
4	*CHO + H_2O + H^+ + e^-→ *OCH_2 + H_2O	-1.277
5	*OCH_2 + H_2O + H^+ + e^- → *OCH_3 + H_2O	0.294
6	*OCH_3 + H_2O + H^+ + e^- → *CH_3OH + H_2O	-1.128
	*OCH_3 + H_2O + H^+ + e^- → *O + CH_4 + H_2O	-1.621
7	*O + CH_4 + H_2O + H^+ + e^- → *OH + CH_4 + H_2O	0.038
	*CH_3OH + H_2O + H^+ + e^- → *OH + CH_4 + H_2O	-0.455
8	*OH + CH_4 + H_2O + H^+ + e^-→ * + CH_4 + 2H_2O	-0.064

Table S10. Gibbs free energy change for each protonation step of V-HAB electrocatalytic CO_2 reduction.

$n(H^+ + e^-)$transferred	Chemical reaction equation	ΔG
1	$* + CO_2 + H^+ + e^- \rightarrow *COOH$	-0.803
	$* + CO_2 + H^+ + e^- \rightarrow *OCHO$	-0.713
2	$*COOH + H^+ + e^- \rightarrow *CO + H_2O$	-0.562
	$*OCHO + H^+ + e^- \rightarrow *OCHOH$	-1.404
3	$*CO + H_2O + H^+ + e^- \rightarrow *CHO + H_2O$	0.453
	$*CO + H_2O + H^+ + e^- \rightarrow *COH + H_2O$	1.523
	$*OCHOH + H^+ + e^- \rightarrow *CHO + H_2O$	1.205
	$*OCHOH + H^+ + e^- \rightarrow *OCH + H_2O$	0.271
4	$*OCH + H_2O + H^+ + e^- \rightarrow *OCH_2 + H_2O$	-0.677
5	$*OCH_2 + H_2O + H^+ + e^- \rightarrow *OCH_3 + H_2O$	-0.359
6	$*OCH_3 + H_2O + H^+ + e^- \rightarrow *CH_3OH + H_2O$	-0.001
	$*OCH_3 + H_2O + H^+ + e^- \rightarrow *O + CH_4 + H_2O$	-0.271
7	$*O + CH_4 + H_2O + H^+ + e^- \rightarrow *OH + CH_4 + H_2O$	0.189
	$*CH_3OH + H_2O + H^+ + e^- \rightarrow *OH + CH_4 + H_2O$	-0.080
8	$*OH + CH_4 + H_2O + H^+ + e^- \rightarrow * + CH_4 + 2H_2O$	0.079
	$*CH_3 + 2H_2O + H^+ + e^- \rightarrow * + CH_4 + 2H_2O$	-0.225

Table S11. Gibbs free energy change for ench protonation step of Cu-HAB electrocatalytic CO_2 reduction.

$n(H^+ + e^-)$transferred	Chemical reaction equation	ΔG
1	$* + CO_2 + H^+ + e^- \rightarrow *COOH$	0.162
	$* + CO_2 + H^+ + e^- \rightarrow *OCHO$	-0.742
2	$*COOH + H^+ + e^- \rightarrow *CO + H_2O$	-0.383
	$*OCHO + H^+ + e^- \rightarrow *OCHOH$	-0.564
3	$*CO + H_2O + H^+ + e^- \rightarrow *CHO + H_2O$	0.178
	$*CO + H_2O + H^+ + e^- \rightarrow *COH + H_2O$	0.751
	$*OCHOH + H^+ + e^- \rightarrow *CHO + H_2O$	1.264
	$*OCHOH + H^+ + e^- \rightarrow *OCH + H_2O$	2.111
	$*CO + H_2O \rightarrow * + CO + H_2O$	0.860
	$*OCHOH \rightarrow * + HCOOH$	1.223
4	$*CHO + H_2O + H^+ + e^- \rightarrow *OCH_2 + H_2O$	-0.809
5	$*OCH_2 + H_2O + H^+ + e^- \rightarrow *OCH_3 + H_2O$	-0.043
	$*OCH_2 + H_2O + H^+ + e^- \rightarrow * + HCHO + H_2O + H^+ + e^-$	0.306
6	$*OCH_3 + H_2O + H^+ + e^- \rightarrow *CH_3OH + H_2O$	-1.258
	$*OCH_3 + H_2O + H^+ + e^- \rightarrow *O + CH_4 + H_2O$	1.175
7	$*CH_3OH + H_2O + H^+ + e^- \rightarrow *OH + CH_4 + H_2O$	0.181
	$*CH_3OH + H_2O \rightarrow * + CH_3OH + H_2O$	0.705
8	$*OH + CH_4 + H_2O + H^+ + e^- \rightarrow * + CH_4 + 2H_2O$	-0.714

Table S12. Gibbs free energy change for each protonation step of Cr-HAB electrocatalytic CO_2 reduction.

n(H^++e^-)transferred	Chemical reaction equation	ΔG
1	* + CO_2 + H^+ + e^- → *COOH	-0.572
	* + CO_2 + H^+ + e^- → *OCHO	-1.344
2	*COOH + H^+ + e^- → *CO + H_2O	-0.276
	*OCHO + H^+ + e^- → *OCHOH	-0.083
3	*CO + H_2O + H^+ + e^- → *CHO + H_2O	0.270
	*CO + H_2O + H^+ + e^- → *COH + H_2O	1.427
	*OCHOH + H^+ + e^- → *CHO + H_2O	0.848
	*OCHOH + H^+ + e^- → *OCH + H_2O	2.308
4	*CHO + H_2O + H^+ + e^- → *OCH_2 + H_2O	-0.105
5	*OCH_2 + H_2O + H^+ + e^- → *OCH_3 + H_2O	-1.211
	*OCH_2 + H_2O + H^+ + e^- → * + HCHO + H_2O+H^++e^-	0.562
6	*OCH_3 + H_2O + H^+ + e^- →*CH_3OH + H_2O	0.270
	*OCH_3 + H_2O + H^+ + e^- →*O + CH_4 + H_2O	-0.856
7	*O + CH_4 + H_2O + H^+ + e^- → *OH + CH_4 + H_2O	-0.212
	*CH_3OH + H_2O + H^+ + e^- → *OH + CH_4 + H_2O	-1.339
	*CH_3OH + H_2O → * + CH_3OH + H_2O	0.803
8	*OH + CH_4 + H_2O + H^+ + e^- → * + CH_4 + $2H_2O$	0.495
